# Supplementary figures and images for: Spatial variations of soil seed banks in Shanghai’s urban wasteland: a gradient analysis of urbanization effects
Source: PeerJ. 2024 Dec 23;12:e18764. doi: 10.7717/peerj.18764 (PMC11670764; doi:10.7717/peerj.18764)

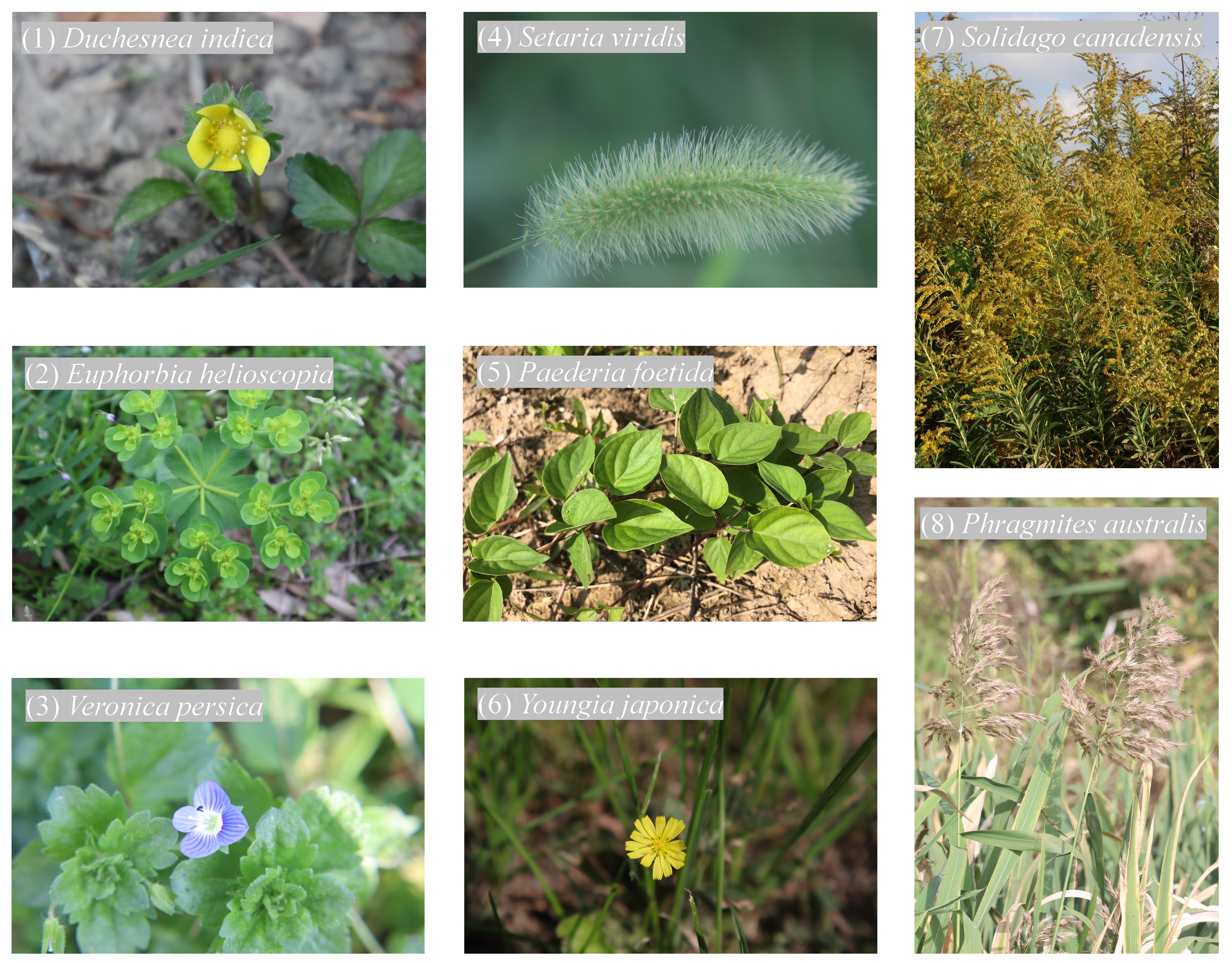

Supplement: Supplemental Information 2 — (1) Procumbent; (2) Rosette; (3) Branched; (4) Tussock; (5) Climbing or liane; (6) Pseudo-rosette; (7) Partial rosette; (8) Erect. 1-5 belong to small growth form and 6–8 belong to large growth form. Photos by Luo Xinyi and Gao Zhiwen. [file peerj-12-18764-s002.png]
