# Supplementary material for: Spatial variations of soil seed banks in Shanghai’s urban wasteland: a gradient analysis of urbanization effects
Source: PeerJ. 2024 Dec 23;12:e18764. doi: 10.7717/peerj.18764 (PMC11670764; doi:10.7717/peerj.18764)
Supplement: Supplemental Information 3 — 1Growth type: p, Procumbent; r, Rosette; b, Branched; t, Tussock; l, Climbing or liane; ps, Pseudo-rosette; pr, Partial rosette; e, Erect. 2Season: spring/autumn, the specie only germinated in season of spring or autumn; both, the specie germinated in both seasons. [file peerj-12-18764-s003.docx]

| Species | Source type | Life form | Growth type^1^ | Dispersal modes | Season^2^ |
| --- | --- | --- | --- | --- | --- |
| *Veronica persica* | exotic | winter annual | b | Autochory | both |
| *Trifolium repens* | exotic | perennial | p | Autochory | autumn |
| *Echinochloa crusgalli* | exotic | summer annual | t | Hydrochory | both |
| *Polypogon fugax* | native | winter annual | t | Zoochory | both |
| *Astragalus melilotoides* | exotic | summer annual | e | Autochory | spring |
| *Potentilla supina* | native | winter annual | b | Autochory | both |
| *Plantago asiatica* | native | perennial | r | Hydrochory | autumn |
| *Rumex dentatus* | native | perennial | ps | Autochory | autumn |
| *Lepidium didymum* | exotic | winter annual | p | Zoochory | autumn |
| *Cirsium arvense var.integrifolium* | native | winter annual | pr | Anemochory | autumn |
| *Cardamine hirsuta* | exotic | winter annual | ps | Autochory | both |
| *Ludwigia prostrata* | exotic | summer annual | e | Autochory | both |
| *Elymus kamoji* | native | perennial | t | Anemochory | autumn |
| *Stellaria media* | native | winter annual | b | Autochory | spring |
| *Trigonotis peduncularis* | native | winter annual | r | Autochory | spring |
| *Setaria viridis* | native | summer annual | t | Anemochory | both |
| *Cynodon dactylon* | native | perennial | t | Autochory | both |
| *Rorippa indica* | native | winter annual | ps | Autochory | spring |
| *Eragrostis pilosa* | native | summer annual | t | Anemochory | spring |
| *Youngia japonica* | native | winter annual | ps | Anemochory | spring |
| *Glechoma longituba* | native | summer annual | e | Anemochory | autumn |
| *Paederia foetida* | native | perennial | l | Autochory | autumn |
| *Solidago canadensis* | exotic | perennial | pr | Anemochory | both |
| *Crepidiastrum sonchifolium* | native | winter annual | p | Anemochory | autumn |
| *Alopecurus aequalis* | native | winter annual | t | Zoochory | spring |
| *Sonchus oleraceus* | exotic | winter annual | pr | Anemochory | both |
| *Ixeris polycephala* | exotic | perennial | pr | Hydrochory | spring |
| *Galium spurium* | native | winter annual | l | Autochory | both |
| *Chenopodium album* | native | summer annual | e | Anemochory | both |
| *Eclipta prostrata* | exotic | summer annual | e | Anemochory | both |
| *Paspalum conjugatum* | exotic | perennial | t | Hydrochory | spring |
| *Solanum nigrum* | exotic | summer annual | b | Zoochory | both |
| *Phragmites australis* | native | perennial | e | Anemochory | both |
| *Cynanchum rostellatum* | native | perennial | l | Autochory | autumn |
| *Humulus scandens* | exotic | summer annual | l | Anemochory | both |
| *Aristolochia debilis* | native | perennial | l | Zoochory | autumn |
| *Digitaria sanguinalis* | native | summer annual | t | Anemochory | both |
| *Dichondra micrantha* | native | perennial | p | Autochory | autumn |
| *Artemisia mongolica* | native | perennial | pr | Autochory | both |
| *Medicago polymorpha* | exotic | winter annual | p | Autochory | both |
| *Eleusine indica* | native | summer annual | t | Autochory | both |
| *Achyranthes bidentata* | native | perennial | e | Zoochory | both |
| *Sagina japonica* | native | summer annual | b | Autochory | spring |
| *Leptochloa chinensis* | native | summer annual | t | Anemochory | both |
| *Ipomoea nil* | exotic | summer annual | l | Anemochory | both |
| *Torilis scabra* | native | winter annual | ps | Zoochory | autumn |
| *Celosia argentea* | exotic | summer annual | e | Autochory | autumn |
| *Abutilon theophrasti* | exotic | summer annual | e | Autochory | spring |
| *Cerastium glomeratum* | exotic | winter annual | b | Autochory | both |
| *Lactuca sibirica* | native | winter annual | pr | Autochory | autumn |
| *Duchesnea indica* | native | perennial | p | Zoochory | autumn |
| *Polygonum hydropiper* | native | summer annual | b | Hydrochory | both |
| *Persicaria lapathifolia* | native | summer annual | e | Autochory | both |
| *Cyperus iria* | native | summer annual | t | Autochory | spring |
| *Hydrocotyle sibthorpioides* | native | winter annual | ps | Anemochory | both |
| *Sesbania cannabina* | exotic | summer annual | e | Autochory | both |
| *Acalypha australis* | native | summer annual | e | Autochory | both |
| *Mazus pumilus* | native | summer annual | ps | Zoochory | spring |
| *Polygonum plebeium* | native | summer annual | p | Hydrochory | autumn |
| *Alternanthera philoxeroides* | exotic | perennial | b | Autochory | both |
| *Chenopodium ficifolium* | native | summer annual | e | Autochory | both |
| *Erigeron canadensis* | exotic | summer annual | pr | Zoochory | both |
| *Glycine soja* | native | summer annual | l | Hydrochory | both |
| *Daucus carota* | exotic | winter annual | pr | Anemochory | both |
| *Geranium carolinianum* | exotic | summer annual | b | Autochory | both |
| *Erigeron annuus* | exotic | summer annual | pr | Zoochory | both |
| *Cyperus difformis* | native | summer annual | t | Autochory | both |
| *Ipomoea purpurea* | exotic | summer annual | l | Autochory | spring |
| *Poa annua* | native | winter annual | e | Hydrochory | both |
| *Euphorbia helioscopia* | exotic | winter annual | r | Zoochory | autumn |
| *Vicia sativa subsp. Nigra* | native | winter annual | l | Autochory | both |
| *Amaranthus viridis* | exotic | summer annual | e | Autochory | both |
| *Oxalis corniculate* | native | perennial | r | Autochory | Autumn |
| *Parthenocissus tricuspidata* | native | summer annual | b | Zoochory | spring |
